# MicroRNAs and Other Small RNAs in Liquid Biopsies as Biomarkers for Early Detection of Colorectal Cancer

**DOI:** 10.3390/ijms27167095

**Published:** 2026-08-07

**Authors:** Natalia Navarro, Javier Gómez-Matas, Carla Di Battista, Meritxell Gironella

**Affiliations:** 1 Gastrointestinal & Pancreatic Oncology Group, Fundació de Recerca Clínic Barcelona—Institut d’Investigacions Biomèdiques August Pi i Sunyer, University of Barcelona, Centro de Investigación Biomédica en Red de Enfermedades Hepáticas y Digestivas (CIBERehd), 08036 Barcelona, Spain; nnavarro@recerca.clinic.cat (N.N.); dibattista@recerca.clinic.cat (C.D.B.); 2 Department of Experimental Pathology, Institute of Biomedical Research of Barcelona-Spanish National Research Council (IIBB-CSIC), 08036 Barcelona, Spain

**Keywords:** colorectal cancer, advanced adenoma, CRC screening, miRNA, liquid biopsy

## Abstract

Early detection of colorectal cancer (CRC) is a major determinant of patient prognosis, as survival strongly depends on disease stage at diagnosis. Despite advances in screening programs, a significant proportion of CRC cases are still diagnosed at advanced stages, underscoring the need for improved early detection strategies. Most sporadic CRCs arise through the adenoma–carcinoma sequence over 10 to 15 years, providing a window for the detection of premalignant lesions, such as advanced adenomas. Current screening approaches are based on colonoscopy or its combination with stool-based tests. Although colonoscopy is the gold standard, it is an invasive technique with high associated costs and limited patient compliance. Stool-based tests are non-invasive and more widely accepted but lack specificity and sufficient sensitivity for detecting premalignant lesions. In this context, liquid biopsies have emerged as a promising minimally invasive alternative for identifying tumor-derived biomarkers in biological fluids such as blood or stool. Small non-coding RNAs (sncRNAs), and particularly microRNAs (miRNAs), have gained considerable attention as non-invasive biomarkers for their highly stability, resistance to handling conditions, and reliable quantification even in low-input samples. Single miRNAs and miRNA signatures detected in biofluids and combined with clinical parameters have shown promise for CRC detection. However, their utility for detecting advanced adenomas remains insufficiently characterized. Further validation in large, independent cohorts and standardization of analytical methods are required before their clinical implementation. Despite these challenges, sncRNA-based liquid biopsies represent a promising approach for improving early detection of CRC and, consequently, its prognosis.

## 1. The Importance of Detecting Colorectal Cancer Earlier

### 1.1. Colorectal Cancer Overview

Colorectal cancer (CRC) is the third most commonly diagnosed cancer worldwide (in males and females) and the second leading cause of cancer-related death. This translates to more than 1.9 million new cases per year and around 900,000 deaths per year worldwide [1,2]. Moreover, the incidence of CRC is predicted to increase to 2.5 million new cases per year by 2035 as a consequence of changes in diet and lifestyle, such as increasing obesity and sedentary habits [1,3].

The 5-year relative survival for CRC is currently around 65% and has improved during the last decades, reflecting both implementation of screening programs and advances in surgical techniques and therapies. However, the prognosis of CRC patients is still strongly dependent on the stage of the disease at diagnosis, as early detection of CRC is the most important factor decreasing mortality. Thus, whereas patients with localized disease have a 5-year relative survival rate of above 90%, survival rates are around 15% for patients with distant metastases [4,5,6]. In addition, patients with advanced disease are more prone to long-term side effects (due to more aggressive treatments) and relapse [7]. Altogether, this emphasizes the importance of improving screening programs for detecting CRC at early stages or even at premalignant stages.

Most sporadic CRCs arise from a polyp, following the adenoma–carcinoma sequence. This process starts with aberrant cells in the bowel crypts evolving into neoplastic precursor lesions (dysplastic adenomas are the most frequent) and eventually into a colorectal malignancy. This process is estimated to last between 10 and 15 years, providing a window of time to screen for and detect precursor lesions [6,8]. Once the malignant transformation has occurred, the progression of the disease is a very complex process in which many factors are involved, including the interaction of tumor cells with their environment (neighbor and immune cells [9,10,11], extracellular matrix [12] and gut microbiota [13,14]), tumor genome stability, and tumor heterogeneity [15,16,17]. Determining the exact time-lapse between CRC onset and metastatic dissemination is still extremely challenging, and studies using phylogenetic reconstruction and computational models have reported both late and early metastatic dissemination. Until recently, it was assumed that metastatic capacities appear late after the accumulation of somatic mutations in tumor cells. However, recent studies suggest that it can occur early, while the primary CRC is still clinically undetectable, and years before the diagnosis [18,19,20,21]. This again underscores the importance of detecting CRC at the earliest possible stage and implementing screening programs for high-risk, but also average-risk, subjects.

Currently, most CRC screening programs recommend the participation of adults over a certain age (usually over 45 or 50 years) and are based on colonoscopy alone or in combination with stool-based tests (primarily fecal occult blood test, FOBT, or fecal immunochemical test, FIT). Colonoscopy is the gold standard technique since it has high sensitivity and specificity for detecting CRC and precancerous lesions [22,23]. However, it still has drawbacks, including high associated costs, requirement of experienced endoscopists, risk of complications (e.g., bleeding and reaction to anesthesia), and patient discomfort during bowel preparation and examination, which leads to lower compliance in comparison to alternative non-invasive methods [22,24,25,26]. Stool-based tests are cheaper and non-invasive and show higher compliance than colonoscopy, but have low sensitivity, especially regarding the detection of premalignant lesions, and low specificity requiring confirmation by colonoscopy [23,27,28]. Since CRC is a preventable and curable disease when diagnosed early, the optimization of non-invasive tools to improve early detection is still a pending clinical need.

### 1.2. Liquid Biopsies for Early Detection of Colorectal Cancer

Liquid biopsies consist of the collection and analysis of different biological fluids to determine the presence/absence of tumor cells or certain tumor characteristics. These approaches provide advantages over traditional biopsies, since they are non-invasive, produce minimal discomfort to the patient, and have low associated costs and risks. In addition, they could be applied for multiple purposes, such as cancer diagnosis, stratification of patients, prediction of treatment response, or relapse monitoring [29,30]. Improvements in the isolation of tumor-related molecules from different sources and in analytical technologies make liquid biopsies a promising tool for tumor precision medicine. In the context of CRC, several studies have reported the detection of circulating tumor cells (CTCs), circulating tumor DNA (ctDNA), and circulating tumor RNA (ctRNA) in different fluids such as blood, stool, or urine [31,32,33] and have centered their efforts on establishing biomarkers for CRC early detection.

The arrival of cancer cells or cancer-derived nucleic acids at different compartments, even far from the primary tumor, is possible due to different mechanisms. On one hand, primary tumor cells can acquire invasive capacities and infiltrate surrounding tissues, eventually reaching the blood or lymphatic vessels [34]. Additionally, tumors tend to have a fast turnover of cells and continuously exhibit cell death (by apoptosis, autophagy, necroptosis, etc.) to some degree. When undergoing cell death, membranes loss their integrity, and nucleic acids can be released into the cell environment [35,36]. Finally, extracellular vesicles containing nucleic acids can also be released by tumor cells into the extracellular space and eventually reach the circulation or other compartments [37]. Blood, including plasma or serum, is the preferred and most explored biological fluid for liquid biopsies, since there is solid evidence for communication between tumors and the circulation. Moreover, blood extraction is routinely used in clinics. In the case of CRC, stool is another interesting biological fluid to consider, since colonocytes and neoplastic cells are constantly exfoliated, and released molecules are in direct contact with the intestinal lumen. In fact, stool-based tests are currently used in CRC screening programs, despite the above-mentioned disadvantages [38]. Lastly, but less explored, other biological fluids such as urine, saliva, or ascitic fluid are also candidates for CRC liquid biopsies, since tumor-derived material has been also found in them [39,40,41].

Although different tumor-derived molecules have been reported in different biofluids from CRC patients, this review will focus on the potential diagnostic value of small non-coding RNAs (sncRNAs). These are short non-coding fragments of RNA that, in contrast to long RNA fragments, are highly stable. It has been demonstrated that they are quite resistant to degradation at room temperature for short periods of time and to manipulating conditions such as freeze-thaw cycles [42]. All these characteristics make them interesting candidates for implementation in clinical practice as non-invasive biomarkers for early detection of CRC.

## 2. Small Non-Coding RNAs as Biomarker Candidates for Early Detection of Colorectal Cancer

### 2.1. Small Non-Coding RNAs Overview

Recent advances in RNA sequencing techniques have allowed the discovery of new functional RNA molecules in what was known as “junk” DNA. These RNAs can be further classified by length as small non-coding RNAs (approximately 20–300 nucleotides) or long non-coding RNAs (>300 nucleotides). SncRNAs are RNA transcripts of less than 300 nucleotides that do not encode proteins. However, they perform a key role in the regulation of other RNA molecules, and their influence is spread across several biological pathways [43]. Moreover, sncRNAs have been associated with multiple aspects of cancer, including tumor initiation, metastasis, and drug resistance. More recently, they have been studied as diagnostic and prognostic biomarkers. Within the diverse group of sncRNAs, different RNA species have been described, including microRNAs, PIWI-interacting RNAs, transfer RNAs, and small nucleolar RNAs [44,45,46] (Table 1), whose role as potential CRC diagnostic markers will be discussed in this review.

MicroRNAs (miRNAs) are the most well-characterized sncRNAs. They are highly evolutionary conserved, single-stranded RNA sequences of 20 to 24 nucleotides that regulate gene expression at the post-transcriptional level. Most miRNA coding sequences are located at intronic regions or clustered in miRNA loci and generate a hairpin structure that is processed and exported to the cytoplasm, where the mature miRNA form can exert its regulatory functions [46,47,48,49]. The canonical function of miRNAs is post-transcriptional gene regulation. Mature miRNAs can interact with messenger RNA (mRNA) sequences and, depending on complementarity between the mRNA and miRNA, this interaction results in cleavage of the mRNA sequence or in its translational repression. This process can be affected by several factors, and even miRNA-mediated up-regulation has been proposed [50,51].

PIWI-interacting RNAs (piRNAs) were more recently described and show more sequence diversity than miRNAs. They are single-stranded RNA sequences of 24 to 32 nucleotides that are generated from single-stranded precursor transcripts. The primary described function of piRNAs is to silence transposable elements and viral sequences, especially those participating in the maintenance of germ and stem cells. However, there is emerging evidence that they can also modulate the translation of protein-coding transcripts, and they have also been associated with gene regulation, epigenetic programming, rearrangement of DNA, modulation of chromatin state and accessibility, and mRNA turnover [52,53]. PiRNAs seem to interact with mRNA and regulate its translation by the same base-pairing rules as miRNAs, although their vaster sequence diversity makes their repertoire of targets and cellular roles even larger [54,55].

Transfer RNAs (tRNAs) are molecules that serve as codon adaptors during ribosomal protein synthesis. Mature tRNAs are sequences of 70 to 93 nucleotides, structured in a complex tridimensional structure with four stem-loops that enable the addition of amino acids during protein synthesis [56,57]. In addition to directly mediating protein synthesis, tRNAs also regulate translation efficiency via tRNA abundance, which, in turn, can modulate many cellular processes and regulate gene expression by other mechanisms, such as tRNA nuclear translocation and post-translational modifications [57]. Finally, there is a recent consensus accepting the existence of stable tRNA breakage products (fragments from 14 to 50 nucleotides), called tRNA-derived fragments (tRFs), which might be involved in many cellular functions. Depending on the cleavage point, tRFs can come from different parts of the tRNA structure: 5′-tRF, 3′-tRF, and inter-tRF [57,58,59].

Finally, small nucleolar RNAs (snoRNAs) are a family of 60- to 300-nucleotide-long single-stranded RNAs. They can be classified in two categories depending on their structure: H/ACA-box and C/D-box. These two categories of snoRNA bind to two different ribonucleoprotein complexes to perform different functions: H/ACA-box snoRNAs perform pseudouridylation of specific nucleotides, and C/D-box snoRNAs guide 2′-O-methylation of rRNA to allow for its maturation. Beyond their canonical functions of processing and post-transcriptional modification of ribosomal RNA, recent studies also showcase that snoRNAs are involved in RNA expression and can affect several biological pathways [60,61,62,63].

SncRNAs have been related to both physiological and pathological conditions, including CRC [64]. Dysregulation in the expression of sncRNAs has been extensively demonstrated in many tumor types, as they are differentially expressed in tumor tissues or other biological fluids. Thus, studies have concluded that they can reflect the presence of the tumor, pathological and clinical features (such as tumor grade or clinical stage), or patient outcome [52,65,66,67]. In addition, sncRNAs are more stable and resistant to storage and handling conditions than other species of RNAs, and they can be detected with high specificity and reproducibility, even in low-input samples [68,69,70,71,72]. Altogether, these features highlight their potential as non-invasive biomarkers for cancer diagnosis or prognosis and predictors of treatment response.

### 2.2. MicroRNAs in Plasma or Serum for CRC Detection

The first studies reporting miRNA in blood were published in 2008 and detected placental miRNAs in maternal plasma [73]. Since then, several groups have been working on the detection of miRNAs in blood for many purposes, including cancer diagnosis. In the context of CRC, the study of miRNAs in circulation has been mainly focused on the detection of malignant tumors, but, interestingly, some studies have included premalignant lesions such as advanced adenomas. These lesions can be easily removed by colonoscopy, thus avoiding progression to CRC. In fact, to date several miRNAs have been detected in plasma or serum samples from patients with CRC or advanced adenomas (Table 2); the members of the miR-17-92 cluster and miR-21 are the most reported ones.

The polycistronic miR-17-92 cluster, also known as oncomiR-1, is an RNA transcript that has a remarkable role in the regulation of cellular processes, such as the cell cycle, proliferation, and apoptosis, and whose dysregulation has been reported in several cancers, including CRC [74,75]. This cluster encodes a set of six tandem miRNAs (miR-17, miR-18a, miR-19a, miR-19b, miR-20a, and miR-92a) that are co-transcribed. Depending on the cellular context, the relative abundance of the mature form of each of these miRNAs can differ and provide information about certain pathological conditions, such as the presence of tumor cells [76,77]. Ng et al. described for the first time overexpression of circulating miR-17-3p and miR-92a in plasma from patients with CRC compared to healthy individuals and suggested the use of these miRNAs as biomarkers for the detection of CRC, with AUCs of 0.717 and 0.885, respectively [78]. Later on, the role of miR-92a as a biomarker for CRC was corroborated by other publications validating their use in plasma or serum samples, with AUCs ranging between 0.809 and 0.871 [79,80,81]. In addition, the role of the other members of this cluster (miR-18a, miR-19a, miR-19b, and miR-20) has also been addressed by other groups, confirming that they are up-regulated in plasma samples from patients with CRC and that they are capable of distinguish patients with CRC from healthy subjects (AUC ranging from 0.682 to 0.849) [80,82,83,84]. Altogether, these results suggest that members of the miR-17-92a cluster are promising candidates for early detection of CRC. Nevertheless, up-regulation in premalignant lesions has only been reported for miR-18a and miR-92a, which in fact were able to segregate patients with advanced adenomas from control subjects, with AUCs between 0.64 and 0.769 [79,82].

Another well-known oncomiR is miR-21, which is overexpressed in many tumors, including CRC [85]. The role of miR-21 as a diagnostic biomarker for CRC has been studied by several research groups, with promising results too. Kanaan et al. showed for the first time that circulating miR-21 in plasma could distinguish CRC patients from controls, with an AUC of 0.91 [86]. Later studies have reached similar conclusions and have corroborated that miR-21 levels in plasma or serum can discriminate patients not only with CRC (AUCs ranging from 0.877 to 0.973) [81,87,88,89,90], but also with advanced adenomas (AUCs ranging from 0.708 to 0.803) [87,91].

Other miRNAs present in plasma or serum samples whose roles as biomarkers for CRC have been reported by more than one research group are miR-27a (AUC 0.697–0.904) [92,93], miR-29a (AUC 0.700–0.844) [79,82], and miR-1290 (AUC 0.718–0.780) [94,95]; miR-29a and miR-1290 are also capable of discerning between healthy subjects and patients with advanced adenomas [79,91,94,95].

**Table 2:** Single miRNAs detected in plasma or serum with potential diagnostic value for colorectal neoplasms.

Reference	Biological Sample	Discovery Method	Validation Sample	Normalizing Control	miRNA	AUC for CRC	AUC for AA	Expression in CRC/AA
Ng et al. 2009 [78]	Plasma	RT-qPCR-based array	CTRL: 50CRC: 90	RNU6B	miR-17-3p	0.717	-	Up
					miR-92	0.885	-	Up
Huang et al. 2010 [79]	Plasma	Based on literature	CTRL: 59 AA: 37 CRC: 100	miR-16	miR-29a	0.844	0.769	Up
					miR-92a	0.838	0.749	Up
Giráldez et al. 2013 [82]	Plasma	miRNA microarray and RT-qPCR	CTRL: 53 AA: 40 CRC: 42	miR-16	miR-18a	0.70–0.80	0.64	Up
					miR-15b	0.70–0.80	-	Up
					miR-19a	0.70–0.80	-	Up
					miR-19b	0.70–0.80	-	Up
					miR-29a	0.70–0.80	-	Up
					miR-335	0.70–0.80	-	Up
Du et al. 2014 [88]	Plasma	Based on literature	CTRL: 49 CRC: 49	Cel-miR-39	miR-21	0.877	-	Up
Fang et al. 2015 [96]	Plasma	Based on literature and public dataset	CTRL: 130 CRC: 111	Cel-miR-39	miR-24	0.839	-	Down
					miR-320a	0.886	-	Down
					miR-423-5p	0.833	-	Down
Liu et al. 2018 [84]	Plasma	Public dataset	CTRL: 50 AA: 50 CRC: 50	RNU6B Cel-miR-39	miR-182	0.891	-	Up
					miR-20a	0.736	-	Up
Liu et al. 2019 [95]	Plasma	Public dataset	CTRL: 30 AA: 50 CRC: 80	RNU6B Cel-miR-39	miR-1290	0.880	0.780	Up
					miR-320d	0.810	0.740	Down
Li et al. 2020 [83]	Plasma	RT-qPCR	CTRL: 585 AA: 19 CRC: 597	RNU6B	miR-20b-5p	0.682	-	Up
					miR-329-3p	0.852	-	Up
					miR-374b-5p	0.914	-	Up
					miR-503-5p	0.734	-	Up
Toiyama et al. 2013 [87]	Serum	Based on literature	CTRL: 53 AA: 43 CRC: 186	Cel-miR-39	miR-21	0.927	0.803	Up
Zheng et al. 2014 [80]	Serum	miRNA sequencing	CTRL: 102 AA: 73 CRC: 117	miR-191-5p RNU6B	miR-19a-3p	0.849	-	Up
					miR-92a-3p	0.871	-	Up
					miR-223-3p	0.890	-	Up
					miR-422a	0.843	-	Down
Imaoka et al. 2016 [94]	Serum	miRNA microarray	CTRL: 57 AA: 56 CRC: 211	Cel-miR-39	miR-1290	0.830	0.718	Up
Vychytilova-Faltejskova et al. 2016 [92]	Serum	Small RNA sequencing	CTRL: 100 CRC: 203	miR-93-5p	miR-142-5p	0.815	-	Up
					miR-23a-3p	0.891	-	Up
					miR-27a-3p	0.697	-	Up
					miR-376c-3p	0.654	-	Up
Guo et al. 2018 [89]	Serum	RT-qPCR	CTRL: 120 AA: 98 CRC: 107	Cel-miR-67	miR-1246	0.681	-	Up
					miR-202-3p	0.815	-	Down
					miR-21-3p	0.878	-	Down
					miR-1229-3p	0.776	-	Up
					miR-532-3p	0.743	-	Up
Sabry et al. 2019 [90]	Serum	Based on literature	CTRL: 101 AA: 35 CRC: 51	RNU6B	miR-21	0.973 *	-	Up
					miR-210	0.934 *	-	Up
					miR-126	0.665 *	-	Down
Li et al. 2021 [97]	Serum	Based on literature	CTRL: 104 CRC: 134	RNU6B	miR-1180-3p	0.956	-	Down
Abo-elela et al. 2023 [93]	Serum	Based on literature	CTRL: 20 NAA: 30 CRC: 70	RNU6B	miR-27a	0.904	-	Up
					miR-133a	0.974	-	Down
					miR-574-3p	0.975	-	Down

Abbreviations: AA, advanced adenoma; AUC, area under the curve; CRC, colorectal cancer; CTRL, healthy control; NAA, non-advanced adenoma; RT-qPCR, real-time quantitative PCR. *AUC discriminating patients with CRC and AA from healthy controls.

Despite detection of single circulating miRNAs seeming to be a promising strategy for identifying patients with advanced colorectal lesions (including AA and CRC), the most encouraging results came from combinations of miRNAs detected in serum or plasma (Table 3). miRNA signatures were able to efficiently identify patients with CRC (with AUCs > 0.95 [80,83,89]) or advanced adenoma (with AUCs > 0.90 [98]). Most of these signatures combined the detection of miR-17-92a cluster members and miR-21 with other miRNAs, while some of them also adjust their predictive model with other patient characteristics such as age, sex, or fecal hemoglobin concentration [99,100]. Special mention goes to Zheng et al., Li et al., and Herreros-Villanueva et al., whose predictive models were able to distinguish CRC from healthy individuals, with AUCs higher than 0.950 [80,83,101]. In the case of Herreros-Villanueva et al., their model also identified patients with advanced adenomas (AUC of 0.910) by detecting miR-17-92a cluster members and miR-335 in plasma samples [101].

**Table 3:** miRNA signatures in plasma or serum with potential diagnostic value for colorectal neoplasms.

Reference	Biological Sample	Discovery Method	Validation Sample	Normalizing Control	Number of miRNAs	miRNA Signature	AUC for CRC	AUC for AA
Huang et al. 2010 [79]	Plasma	Based on literature	CTRL: 59AA: 37 CRC: 100	miR-16	2	miR-29a miR-92a	0.883	0.773
Kanaan et al. 2013 [102]	Plasma	RT-qPCR-based array	CTRL: 26 AA: 16 CRC: 45	RNU6B	2	miR-431 miR-139-3p	0.829	-
					8	miR-532-3p miR-331 miR-195 miR-17 miR-142-3p miR-15b miR-532 miR-652	-	0.868
Giraldez et al. 2013 [82]	Plasma	miRNA microarray and RT-qPCR	CTRL: 53 AA: 40 CRC: 42	miR-16	3	miR19a miR19b miR15b	0.836	-
Fang et al. 2015 [96]	Plasma	Based on literature and public dataset	CTRL: 130 CRC: 111	Cel-miR-39	3	miR-24 miR-320a miR-423-5p	0.899	-
Liu et al. 2018 [84]	Plasma	Publicly available datasets	CTRL: 50 AA: 50 CRC: 50	RNU6B Cel-miR-39	2	miR-182 miR-20a	0.831	-
Liu et al. 2019 [95]	Plasma	Publicly available datasets	CTRL: 30 AA: 50 CRC: 80	RNU6B Cel-miR-39	2	miR-1290 miR-320d	0.910	0.820
Herreros-Villanueva et al. 2019 [101]	Plasma	Based on previous studies	CTRL: 100 AA: 101 CRC: 96	miR-1228 Cel-miR-39	6	miR-15b miR-18a miR-19a miR-19b miR-29a miR-335	0.950	0.910
Li et al. 2020 [83]	Plasma	miRNA microarray and RT-qPCR	CTRL: 585 AA: 19 CRC: 597	RNU6B	4 (+two non-coding RNAs)	miR-20b-5p miR-329-3p miR-374b-5p miR-503-5p	0.954	-
Nakamura et al. 2022 [103]	Plasma	Publicly available dataset	CTRL: 65 CRC: 77	miR-103a-3p	4	miR-193 miR-210 miR-513 miR-628	0.880	-
Liu et al. 2023 [100]	Plasma	Based on literature	CTRL: 103 CRC: 97	miR-16	3 (+age, sex, smoking, drinking, and obesity status)	miR-29a miR-125b miR-145	0.710	-
Wang et al. 2014 [104]	Serum	Based on literature	CTRL: 59 CRC: 83	miR-16	6	miR-21 Let-7g miR-31 miR-92a miR-181b miR-203	0.923	-
Zheng et al. 2014 [80]	Serum	miRNA sequencing	CTRL: 102 AA: 73 CRC: 117	RNU6B miR-191-5p	4	miR-19a-3p miR-92a-3p miR-223-3p miR-422a	0.951	0.765
Vychytilova-Faltejskova et al. 2016 [92]	Serum	Small RNA sequencing	CTRL: 100 CRC: 203	miR-93-5p	4	miR-142-5p miR-23a-3p miR-27a-3p miR-376c-3p	0.922	-
Guo et al. 2018 [89]	Serum	RT-qPCR	CTRL: 120 AA: 98 CRC: 107	Cel-miR-67	5	miR-1246 miR-202-3p miR-21-3p miR-1229-3p miR-532-3p	0.960	-
Marcuello et al. 2019 [99]	Serum	Based on previous studies	CTRL: 80 AA: 74 CRC: 59	miR-1228	6 (+FIT)	miR-15b miR-18a miR-19a miR-19b miR-29a miR-335	0.880	0.810

Abbreviations: AA, advanced adenoma; AUC, area under the curve; CRC, colorectal cancer; CTRL, healthy controls; FIT, fecal hemoglobin concentration; RT-qPCR, real-time quantitative PCR.

At this point, it is important to note that, although promising results have been reported, with several studies achieving excellent AUC values, only a few studies included in this review have externally validated their findings in a second, independent cohort. This is the case for the studies conducted by Giráldez et al. [82], Vychytilova-Faltejskova et al. [92], Herreros-Villanueva et al. [101], and Guo et al. [89]. Moreover, none of the studies have reached the clinical validation stage. This is particularly relevant, and the reported AUC values should be interpreted with caution, as they may overestimate real-world performances, which highlights a window for improvement that still exists in the field.

In addition, it is noteworthy that almost only members of the miR-17-92a cluster, miR-21, and miR-29a have been consistently identified as miRNAs with the best performances across independent studies. Several factors may underlie this perception of limited reproducibility, including technical aspects such as differences in discovery platforms, validation methodologies, and normalizing controls. However, biological heterogeneity between cohorts may also play an important role and be poorly considered by many studies in the field. CRC tumors exhibit distinct molecular and anatomical characteristics that influence expression profiles, such as chromosomal instability status [105] or tumor location [106], which in turn may influence circulating miRNA signatures. These factors could partially explain discrepancies among studies and should be more carefully considered for the future validation of biomarkers.

### 2.3. MicroRNAs in Stool for CRC Detection

Soon after miRNAs were detected in the circulation, studies reporting their presence in stool samples were published, and miRNAs were proposed as promising biomarkers also in this biofluid. In the context of CRC diagnosis, Ahmed et al. were the first to demonstrate the feasibility of miRNA detection in stool from patients with CRC, more than a decade ago [107]. Afterwards, other studies performed by Link et al. and Koga et al. corroborated the viability of measuring miRNAs in stool [108,109]. They reported up-regulation of some miRNAs in samples from patients with adenomatous polyps and CRC, such as miR-21, miR-106a, and members of the already mentioned miR-17-92 cluster.

Although the number of studies conducted of stool samples is far less than that of studies performed of blood samples, promising results have been published so far. As a matter of fact, miR-21 and miR-92a (member of the miR-17-92 cluster) continue to be the most reported individual miRNAs in stool from CRC patients. In addition, detection of miR-21 or miR-92a in stool and FIT leftovers was able to distinguish patients with CRC from healthy subjects, with AUCs ranging from 0.620 to 0.780 [110,111,112,113]. However, miR-144* and miR-223 detected in stool showed higher potential to discriminate CRC patients from healthy individuals, with AUCs of 0.829 and 0.820, respectively [114,115]. Other stool miRNAs and their potential as biomarkers are summarized in Table 4.

While detection of single stool miRNAs provides good results, the most promising results come from combinations of miRNAs (Table 5). In this regard, Duran-Sanchon et al. and Pardini et al. were able to efficiently identify patients with CRC (AUC 0.87–0.96) using a stool miRNA signature combined with age, sex, and fetal hemoglobin concentration [111,116,117]. Both were able to detect these miRNA signatures in leftovers from samples used for fetal hemoglobin assessment (FIT). This is remarkable, since stool-based tests such as FIT are currently applied in screening programs, and the use of the same sample for detecting miRNA biomarkers would probably ease their implementation in the clinical setting. Regarding detecting premalignant lesions via fecal miRNAs, few studies have been conducted with this purpose. Nevertheless, Wu et al., Duran-Sanchon et al., and Santos et al. described models that were able to distinguish patients with premalignant lesions (advanced adenomas) from healthy individuals, with AUCs between 0.64 and 0.83 [110,113,117,118].

**Table 4:** Stool miRNAs with potential diagnostic value for colorectal neoplasms.

Reference	Discovery Method	Biological Sample	Validation Sample	Normalizing Control	miRNA	AUC for CRC	AUC for AA	Expression in CRC/AA
Wu et al. 2012 [110]	Based on previous studies and literature	Stool	CTRL: 101 NAA/AA: 57 CRC: 88	Absolute quantification	miR-21	0.640	-	Up
					miR-92a	0.780	-	Up
Wu et al. 2014 [118]	miRNA microarray and RT-qPCR	Stool	CTRL: 109 IBD: 42 AA: 169 CRC: 104	RNU6B	miR-135b	0.790	0.710	Up
Ghanbari et al. 2015 [119]	miRNA microarray and RT-qPCR	Stool	CTRL: 16 CRC: 40	RNU6B	miR-4487	0.700	-	Down
					miR-1295	0.710	-	Down
Zhu et al. 2016 [120]	Based on previous studies	Stool	CTRL: 51 CRC: 80	Balance sample	miR-29a	0.777	-	Down
					miR-223	0.649	-	Down
					miR-224	0.744	-	Down
Duran-Sanchon et al. 2019 [111]	RNA sequencing and RT-qPCR	FIT+ leftovers	CTRL: 217 NAA: 136 AA: 347 CRC: 67	Absolute quantification	miR-130b-3p	0.710	0.690	Up
					miR-21-5p	0.690	0.650	Up
					miR-221-3p	0.700	0.640	Up
					miR-25-3p	0.700	0.650	Up
					miR-27a-3p	0.690	0.650	Up
					miR-29a-3p	0.690	0.640	Up
					miR-335-3p	0.670	0.650	Up
					miR-34a-5p	0.710	0.640	Up
					miR-421	0.770	0.710	Up
Chen et al. 2023 [115]	Based on previous studies	Stool	CTRL: 249 CRC: 94	miR-222	miR-223	0.820	-	Up

Abbreviations: AA, advanced adenoma; AUC, area under the curve; CRC, colorectal cancer; CTRL, healthy control; FIT, fecal immunochemical test; IBD, inflammatory bowel disease; NAA, non-advanced adenoma; RT-qPCR, real-time quantitative PCR.

Again, it is noteworthy that, although some studies reported excellent AUC values, only a few studies included in this review have externally validated their findings in second, independent cohorts. This is the case for the studies conducted by Duran-Sanchon et al. [111,117] and Pardini et al. [116]. Moreover, none of the studies have reached the clinical validation phase. Therefore, these AUC values should be interpreted with caution, as they may overestimate the biomarker’s performance.

Finally, the detection of miRNAs in other biological fluids apart from blood and stool has not been frequently reported. However, miRNAs have been detected in other fluids such as urine, saliva, and peritoneal lavage fluid from CRC patients. Iwasaki et al. reported up-regulation of miR-129-1-3p and miR-566 in urine from patients with CRC compared to healthy subjects and described a combined model that was able to identify patients with CRC, with an AUC of 0.686 [39]. Rapado-González et al. examined the performance of a panel of five miRNAs (miR-186-5p, miR-29a-3p, miR-29c-3p, miR-766-3p, and miR-491-5p) that were up-regulated in the saliva of CRC patients (AUC of 0.744) [40]. Roman-Canal et al. reported the up-regulation of several miRNAs in peritoneal lavage fluid from patients with CRC compared to samples from healthy donors [41]. Although peritoneal lavage would not be the ideal non-invasive liquid biopsy, in that study some miRNAs were able to distinguish patients with CRC from healthy donors, with AUCs above 0.95 (miRNA-199b-5p, miRNA-150-5p, miRNA-29c-5p, miRNA-218-5p, miRNA-99a-3p, miRNA-383-5p, miRNA-199a-3p, miRNA-193a-5p, miRNA-10b-5p, and miRNA-181c-5p). Altogether, these preliminary results suggest that miRNAs in these biological fluids should be further characterized in the context of CRC.

**Table 5:** miRNA signatures in stool with potential diagnostic value for colorectal neoplasms.

Reference	Discovery Method	Biological Sample	Validation Sample	Normalizing Control	Number of miRNAs	miRNA Signature	AUC for CRC	AUC for AA
Duran-Sanchon et al. 2019 [111], 2020 [117]	miRNA sequencing and RT-qPCR	FIT+ leftovers	CTRL: 217 NAA: 136 AA: 347 CRC: 67	Absolute quantification	2 (+FIT, sex, and age)	miR-27a-3p miR-421	0.890	0.70
Chen et al. 2023 [115]	Based on previous studies	Stool	CTRL: 249 CRC: 94	miR-222	4	miR-223 miR-92a miR-16 miR-20a	0.821	-
Pardini et al. 2023 [116]	Small RNA sequencing	Stool	CTRL: 80 CRC: 141	Global mean	5 (+age, sex)	miR-607-5p miR-6777-5p miR-4488 miR-149-3p miR-1246	0.960	-

Abbreviations: AA, advanced adenoma; AUC, area under the curve; CRC, colorectal cancer; CTRL, healthy control; FIT, fecal immunochemical test; NAA, non-advanced adenoma.

### 2.4. Other Small Non-Coding RNAs in Biological Fluids with Potential for CRC Detection

PiRNAs are one of the most commonly explored sncRNAs in circulation for early detection of CRC, apart from miRNAs. Mai et al. demonstrated that piR-54265 plays an oncogenic role in CRC [121]. Using droplet digital PCR (ddPCR), they measured its concentration in serum not only from CRC patients and healthy controls, but also from other digestive cancer patients. The levels of piR-54265 in patients with other digestive cancers were similar to those in cancer-free individuals, but lower than those in patients with CRC, suggesting that piR-54265 could be a CRC-specific biomarker. The AUC was 0.896 for discriminating CRC patients from healthy controls and 0.946 for identifying CRC among other digestive cancers.

A study by Vychytilova-Faltejskova et al. reported that piR-5937 and piR-28876 were down-regulated in serum from a cohort of 403 CRC patients compared to 276 healthy donors [122]. Surprisingly, the expression of these piRNAs presented a decreasing trend in a stage-dependent manner. These piRNAs could act as diagnostic biomarkers for CRC in serum, with AUCs of 0.819 for piR-5927 and 0.726 for piR-28876. However, the combination of these two piRNAs did not yield higher diagnostic potential compared to being analyzed individually.

Sabbah et al. reported an up-regulation of piR-823 in the serum of CRC patients compared to healthy individuals and also in tumor tissues from the same individuals compared to adjacent normal tissue samples [123]. The expression of this piRNA in tissues was significantly associated with advanced tumoral stage and poor differentiation. PiR-823 in serum samples presented an AUC of 0.933 for discriminating patients with CRC from healthy controls.

Wang et al. found up-regulation of piR-020619 and piR-020450 in serum samples from CRC compared to healthy individuals from four different cohorts that also included several other pathologies [124]. After generation of a logistic regression model and validation of the results in an independent cohort, these piRNAs showed high discriminating capacity to detect patients with CRC and AA, with AUCs of 0.883 and 0.779, respectively.

In 2019, Qu et al. detected five piRNAs that were down-regulated in serum samples from CRC patients compared to healthy patient samples: piR-001311, piR-004153, piR-017723, piR017724, and piR-020365 [125]. A diagnostic potential comprising these piRNAs showed capacity to identify patients with CRC, with AUCs ranging from 0.726 to 0.786. The combination of piRNAs in a panel yielded an AUC of 0.854, a diagnostic ability superior to that of CEA and CA19-9 combined.

SNORD1C was up-regulated in serum from CRC patients compared to samples from healthy individuals and patients with benign colorectal pathologies [126]. In that study, SNORD1C presented an AUC of 0.748, which was slightly superior to that of CEA (AUC = 0.715). These two biomarkers combined achieved an AUC of 0.838.

Based high-throughput RNA sequencing of plasma samples, Wu et al. concluded that 5′-tRFs, and more specifically 5′-tRF-GlyGCC, might be involved in the development of CRC [127]. This result was confirmed by RT-qPCR and further analyzed in a different set of plasma samples from CRC patients and healthy controls. The levels of this tRF increased in a stage-dependent fashion but were higher than healthy controls at all stages. The capacity of this tRF to detect CRC was analyzed by ROC curves, yielding an AUC of 0.882, while the combination of these three biomarkers resulted in an improved AUC of 0.926.

Although relatively few scientific studies have explored the role of other sncRNAs in other biological fluids such as stool, emerging evidence highlights their potential clinical value. For instance, Gómez-Matas et al. detected snoRNAs in fecal samples and evaluated their diagnostic performance for CRC. Interestingly, SNORA51, when combined with hemoglobin concentration, age, and sex, generated a predictive model capable of identifying CRC patients with an AUC of 0.866, and those with AA with an AUC of 0.662 [128].

Taken together, the results described in these articles showcase a new source of potential circulating biomarkers for CRC based on species of sncRNAs other than miRNAs (Table 6 and Table 7). The diagnostic potential reported by the authors was superior to that of CEA and CA19-9, two of the most commonly analyzed circulating biomarkers in CRC and other cancers [129].

**Table 6:** Single sncRNAs detected in biofluids with potential diagnostic value for colorectal neoplasms.

Reference	Sample	Discovery Method	Validation Sample	HK	sncRNA	AUC for CRC	AUC for AA	Expression in CRC/AA
Qu et al. 2019 [125]	Serum	Next-generation sequencing	CTRL: 220 CRC: 220	miR-192-5p RNU6B	piR-001311	0.726	-	Down
					piR-004153	0.729	-	Down
					piR-017723	0.756	-	Down
					piR-017724	0.749	-	Down
					piR-020365	0.786	-	Down
Vychtilova-Faltejskova et al. 2018 [122]	Serum	Next-generation sequencing	CTRL: 100 CRC: 179	piR-28131	piR-5937	0.767	-	Down
					piR-28876	0.707	-	Down
Mai et al. 2020 [121]	Serum	Public databases	CTRL: 209 CRC: 725	cel-miR-39	piR-54265	0.896	-	Up
Sabbah et al. 2021 [123]	Serum	Based on literature	CTRL: 75 CRC: 84	RNU6B	piR-823	0.933	-	Up
Wang et al. 2020 [124]	Serum	Next-generation sequencing	CTRL: 140 AA: 40 CRC: 140	piR-001918	piR-020619	0.871	0.701	Up
					piR-020450	0.841	0.689	Up
Liu et al. 2021 [126]	Serum	Based on literature	CTRL: 50 CRC: 122	RNU6B	SNORD1C	0.748	-	Up
Wu et al. 2021 [127]	Plasma	Next-generation sequencing	CTRL: 90 CRC: 105	RNU6B	5′-tRF-GlyGCC	0.882	-	Up

Abbreviations: AA, advanced adenoma; AUC, area under the curve; CRC, colorectal cancer; CTRL, healthy control.

**Table 7:** sncRNA signatures in biofluids with potential diagnostic value for colorectal neoplasms.

Reference	Sample	Discovery Method	Validation Sample	HK	Number of sncRNAs	sncRNA Signature	AUC for CRC	AUC for AA
Vychtilova-Faltejskova et al. 2018 [122]	Serum	Next-generation sequencing	CTRL: 100 CRC: 179	piR-28131	2	piR-28876 piR-5937	0.765	-
Qu et al. 2019 [125]	Serum	Next-generation sequencing	CTRL: 220 CRC: 220	miR-192-5p U6 snRNA	5	piR-001311 piR-004153 piR-020365 piR-017724 piR-017723	0.854	-
Wang et al. 2020 [124]	Serum	Next-generation sequencing	CTRL: 140 CRC: 140 AA: 40	piR-001918	2	piR-020619 piR-020450	0.883	0.779
Gómez-Matas et al. 2024 [128]	FIT + leftovers	Next-generation sequencing	CTRL: 52 AA: 63 CRC: 56	Absolute quantification	1 (+FIT, age, and sex)	SNORA51	0.866	0.662

Abbreviations: AA, advanced adenoma; AUC, area under the curve; CRC, colorectal cancer; CTRL, healthy control.

## 3. Challenges in the Establishment of sncRNAs as Non-Invasive Biomarkers for CRC Early Detection

**Figure 1:**
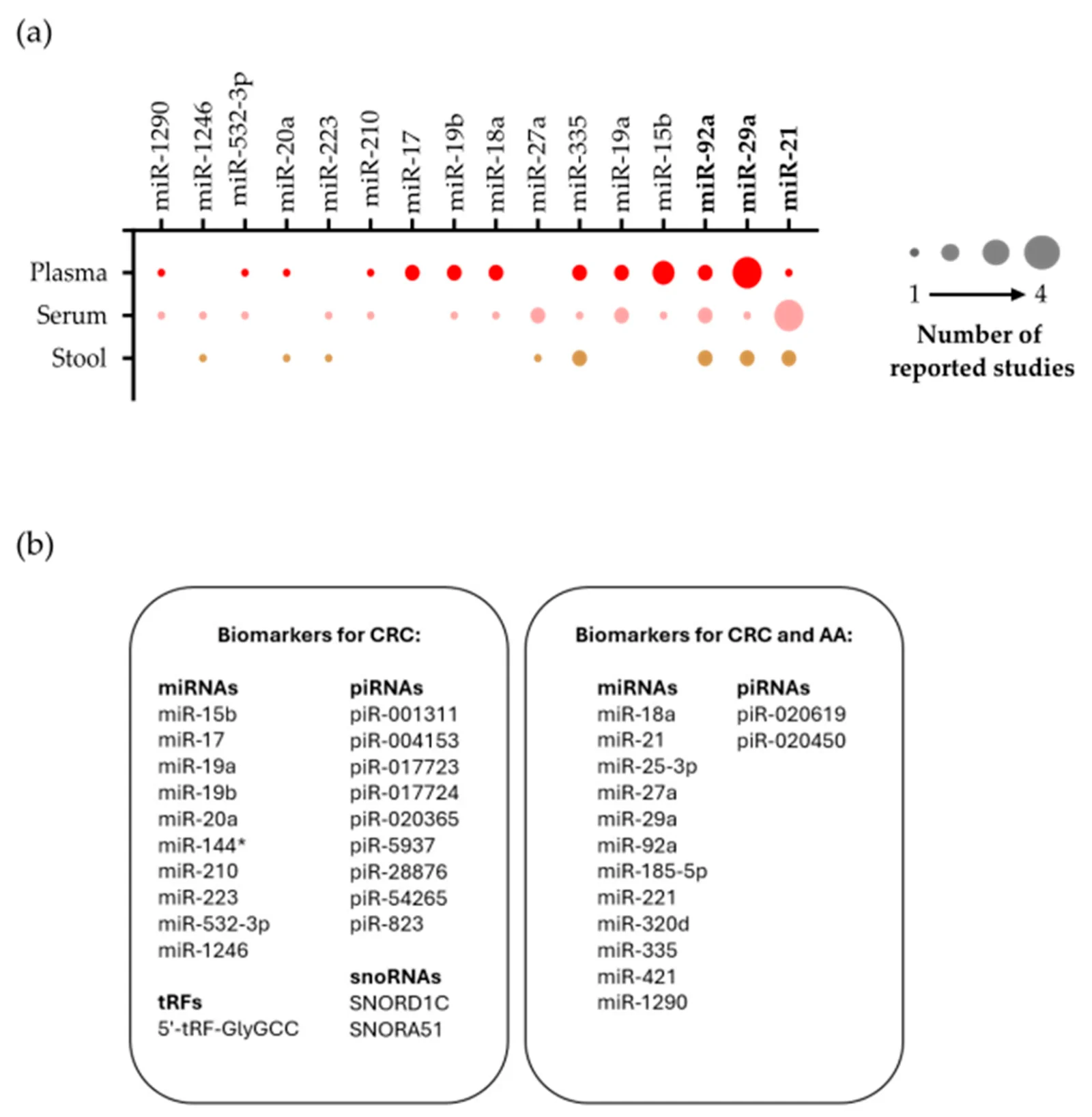
(**a**) List of the miRNAs most frequently reported as candidate biomarkers for CRC detection from different biological sources. The circle size indicates the number of studies that reported each miRNA. (**b**) List of candidate liquid biopsy biomarkers. SncRNAs that have been reported as potential biomarkers for CRC or advanced CRC neoplasm (including CRC and AA) in body fluids. Abbreviations: AA, advanced adenomas;; CRC, colorectal cancer.

After reviewing the published data regarding sncRNAs in the context of CRC screening and diagnosis, miR-21, miR-29a, and miR-92a stood out as the most frequently reported sncRNAs in liquid biopsies (Figure 1a). They emerged as promising non-invasive biomarkers for CRC in blood and stool samples and were able to distinguish CRC patients from healthy subjects, with high diagnostic performances (AUCs > 0.9, Table 2 and Table 4), alone or in combination with other parameters. However, the specificity of these miRNAs for CRC should be further explored, since the expression levels miR-21 and miR-92a, and to a lesser extent miR-29a, are altered in many different situations, which could result into a high number of false positives for a potential diagnostic test based on these miRNAs. miR-21 dysregulation has been documented across a wide spectrum of diseases, particularly in several cancers [85,130] and inflammatory and autoimmune disorders [131,132,133], but also in cardiovascular diseases [134,135], neurological disorders [136], fibrotic diseases, and infections [137], and a similar situation occurs with miR-92a [138,139,140]. These facts result in a lack of disease specificity, which is a major limitation for these miRNAs being used as liquid biopsy biomarkers for a given disease. It is also worth mentioning that miR-92a is abundantly expressed in blood cells [141,142] and is therefore markedly affected by hemolysis, making its circulating levels especially susceptible to pre-analytical variability and potential overestimation. Moreover, miR-21 is highly enriched in circulating immune cells, more than in erythrocytes; thus, it is moderately influenced by hemolysis, but its quantification is partially confounded by immune cell contributions. In addition, they still need to be further characterized in larger populations, and their performance as diagnostic tools has to be compared to the currently used standards for CRC screening (mainly stool-based tests and colonoscopy).

Considerable efforts have been made by Li et al. and Duran-Sanchon et al. [83,114,117], whose studies validated the use of single miRNAs in blood and stool samples, respectively, in large populations. Similarly, miRNA signatures combined with other clinical parameters, such as the ones proposed by Guo et al. and Li et al. in blood samples [83,89] or by Duran-Sanchon et al. and Pardini et al. in stool [116,117], also showed good diagnostic performances (AUCs > 0.90, Table 3 and Table 5) and efficiently identified CRC patients. However, validations in larger and independent cohorts are still necessary, as well as a formal comparison with the gold standard techniques for CRC diagnosis.

In addition, considering the aim of screening programs and the natural course of CRC, detection of advanced adenomas is probably a key determinant that eventually affects CRC incidence and mortality. Despite this, few studies have addressed the detection of premalignant lesions, and more effort is still needed in this regard. Among the studies consulted, again, miR-21, miR-29a, and miR-92a, as well as other members of the miR-17-92 cluster, are promising biomarkers for the detection of both CRC and advanced adenomas in liquid biopsies (Figure 1b). Remarkably, Carter et al. and Herreros-Villanueva et al. were able to characterize a blood-based miRNA signature that identified patients with CRC and advanced adenomas, with good diagnostic performances (AUC > 0.90, Table 3) [98,101], whereas Duran-Sanchon et al. characterized a stool-based miRNA signature (based on miR-421 and miR-27a detection) that detected CRC and advanced adenomas in the largest CRC screening population studied so far [111,117]. Other sncRNAs apart from miRNAs have been reported as diagnostic biomarkers, although the number of studies is far smaller. PiRNAs stand out as the second most reported sncRNA class with diagnostic capacities (AUCs up to 0.933). Yet it is still an immature field lacking standardized databases for annotation, standardized methodologies to analyze them, and validation cohorts, indicating that more studies are needed to elucidate the biomarker potential of these molecules in different biofluids.

Validation of single miRNAs and miRNA signatures in larger populations is still a major issue for the development and establishment of miRNA-based liquid biopsy tests, and other analytical challenges also need to be faced in this field. Indeed, notable heterogeneity in terms of patient selection, sample collection, discovery technologies, and normalization methods have been observed in the reviewed studies.

### 3.1. Patient Selection and Definition of Groups

The consulted studies compared levels of different sncRNAs in different biological fluids from different subject groups, namely patients with CRC, patients with premalignant lesions, and healthy individuals. However, although all these groups were well-represented in some studies that considered all the steps in the physiopathology of CRC, many studies did not include patients with premalignant lesions. Given the well-characterized course of events that lead to CRC (at least for tumors following the adenoma–carcinoma sequence), inclusion of patients with premalignant lesions is important. Finding a biomarker with high sensitivity for detecting premalignant lesions would allow the excision of polyps that are not yet malignant via endoscopic procedures (mostly minor interventions) and would reduce the incidence of CRC and, eventually, CRC mortality. In addition, good representation of the different phases of the disease could increase the power of validation studies. Thus, inclusion of patients with CRC at different stages at the moment of diagnosis should be also considered.

The current CRC screening strategies frequently include stool-based tests, such as FIT, whose results might be another interesting feature to take into consideration. Including individuals with positive and negative FIT values in the study design could be useful not only to evaluate the clinical performance of new biomarkers compared with current screening tests, but also to adjust new predictive models that combine the detection of sncRNAs with epidemiologic data and FIT determinations, such as the models generated by Duran-Sanchon et al. [111,117] and Pardini et al. [116].

Finally, other sources of biological heterogeneity should also be taken into consideration. Factors such as the presence of serrated adenomas, early-onset tumors, tumor location (colon versus rectum, or different colonic segments), and molecular tumor characteristics (e.g., chromosomal instability status or consensus molecular subtypes) remain poorly described and are not often considered in studies within the field. However, these variables may influence gene expression profiles and, consequently, alter circulating RNA signatures [105,106]. These factors could partially explain the discrepancies observed across studies and should be more carefully considered in future validation studies.

### 3.2. Sample Source and Potential Clinical Interferences

Blood, including plasma and serum, and to a lesser extent stool samples have been studied in the context of CRC. In both cases, miRNAs seem to be well preserved and resist storage and manipulating conditions, making these types of samples and biomarkers easy to use in clinical practice. Recently, studies such as those conducted by Duran-Sanchon et al. and Pardini et al. characterized the presence of miRNAs in FIT leftovers [111,116], suggesting a new sample source (remaining stool sample mixed with the FIT buffer). This is noteworthy, since current screening programs commonly involve stool-based tests, such as FIT or FOTB. The combination of miRNA signatures with other epidemiological data or hemoglobin values seems to generate a more sensitive predictive model for the detection of advanced neoplastic lesions. Furthermore, the implementation of a possible diagnostic tool using FIT/FOTB leftovers would be facilitated by the current use of these stool-based tests in routine clinical practice.

While the potential use of miRNA biomarkers in CRC screening programs seems to be very encouraging, it is still limited by technical difficulties and other possible interferences. In blood samples, for instance, hemolysis can occur during sample extraction or preparation and may influence accurate determination of circulating miRNAs, since some of them are also expressed in blood cells. Therefore, it is important to consider that, as certain miRNAs (such as miR-451) are highly abundant in red blood cells, they could serve as markers to detect and discard hemolyzed samples in order to avoid introducing bias [143]. Alternatively, other methods to measure hemolysis could be used for this purpose. Similarly, miRNAs present in platelets in plasma are susceptible to alteration via pre-analytical manipulation, such as sample preparation by centrifugation, and should therefore be carefully considered, as platelet content may influence miRNA levels and compromise reproducibility [144].

On the other hand, clinical features such as the presence of non-malignant lesions (e.g., inflammatory disease) or bacterial intestinal colonization should be considered, since they could interfere with the determination of miRNA expression levels, especially in stool and FIT samples. In fact, there are studies that confirm communication between host miRNAs and the gut microbiota. Several cancer-related bacteria (e.g., *F. nucleatum*, *E. coli,* and *B. fragilis*) have been shown to modulate miRNA levels in CRC cells, while conversely, certain miRNAs can be taken up by bacteria [145]. This interaction between host and microbiota, although still poorly understood, could modify miRNA levels (and presumably the levels of other sncRNAs) and therefore should be taken into account during the validation of non-invasive biomarkers. Finally, dysregulation of miRNAs has been reported in other intestinal diseases. For instance, certain miRNAs mentioned in this review (miR-21, miR-223, and miR-1246, among others) have been associated to other intestinal diseases such as Crohn’s disease and ulcerative colitis [146,147]. Thus, it is important to consider the presence of other pathological entities to define the sensitivity and specificity of CRC biomarkers. As mentioned before, it is also noteworthy that the presence of tumors other than CRC can also modify the sncRNA profile, as is the case for miR-21, which has been proposed as biomarker for different types of cancer, such as breast, pancreatic, and esophageal [85,130,148], as well as for other diseases [131,132,133,134,135,136,137]. Ultimately, there is still room for improvement, but consideration of these possible biases could strengthen the quality of validation studies.

### 3.3. Methodological Issues

Different methodologies are employed for sncRNA discovery and validation. Studies that included a first discovery phase (not based on the literature) mainly used microarray or next-generation sequencing (NGS) techniques to identify and quantify sncRNAs present in tissues or biofluids; and both of these technologies have their own limitations and strengths. On one hand, microarrays allow assessment of the expression of hundreds of sncRNA in a single assay, but the set of sncRNAs that can be detected is limited by the restricted number of probes included in each platform. In contrast, NGS allows analysis and quantification of all the small RNAs present in a sample, without limitation of the number of RNA transcripts to be detected. As a result, NGS can capture, for example, the entire miRNome in a single experiment, making it a powerful tool for biomarker identification. However, NGS remains an expensive technique that requires a high quantity and quality, if possible, of RNA, as well as extensive computational analysis. Also, sncRNAs detected by NGS have to be aligned with sequences annotated in database repositories, making this technique dependent on previously reported knowledge [149,150]. Despite having its own drawbacks, NGS has progressively replaced microarrays in the biomarker discovery field. Technical characteristics of microarrays and NGS may also explain the variability in the published literature. In other words, it is possible that some sncRNAs have been less frequently reported in studies just because they were described and annotated more recently, or were not frequently included in microarrays because they were not yet associated with cancer-related pathways. This technical source of variability has to be added to the intrinsic biological variability of the studies focused on biomarker discovery. The discovery and annotation of sncRNAs, together with the development of techniques and methodologies for their detection and characterization, have progressed substantially over the last two decades. The evaluation of these molecules as novel biomarkers for the detection of advanced colorectal neoplasia has occurred in parallel with these advances, which may partially explain their limited reproducibility and clinical validation. This may have contributed to slow implementation of sncRNA-based biomarkers in clinical practice and have represented an additional barrier to clinical translation so far.

Regarding the validation phase, qRT-PCR has been the preferred detection method so far because of its high sensitivity, specificity, and reproducibility for detecting low levels of RNA species. Reliable measurements with qRT-PCR also depend on the efficiency and quality of the RNA extraction, normalization, and stable reaction conditions. While extraction protocols and reaction conditions are more standardized, normalization of the generated data is still a pending issue. The consulted publications used different methods for normalization, mainly through reference snRNA such as U6 small nuclear RNA-6 (RNU6B), endogenous miRNAs such as miR-16, or exogenous spike-in controls such as Cel-miR-39 or Cel-miR-67. Only a few studies used global normalization (calculated mean of all sncRNAs analyzed) or absolute quantification (understood as extrapolation to a standard curve). Each methodology employed has positive and negative aspects. Both RNU6B and endogenous miR-16 levels have been reported to vary under certain conditions: after freeze-thaw cycles or in highly degraded RNA, and after hemolysis, respectively. Moreover, RNU6B is rarely found in the circulation, or levels are too low to be reliable. In the absence of a good endogenous control for normalization, exogenous controls are sometimes used, which are good for technically normalizing differences due to varying RNA extraction efficiencies and procedures but cannot normalize endogenous variability [143,151]. All these aspects are still challenging and should be hence taken into consideration for successful validation of biomarkers and their application in clinical practice.

Finally, this review has focused on a broad description of free-circulating sncRNAs in various biological sources (e.g., blood, feces). However, it has not addressed their presence within extracellular vesicles (EVs) or exosomes, which may represent valuable sources of biomarkers for the detection of advanced colorectal neoplasia [152,153,154]. Pre-analytical and analytical variables affecting EV-derived biomarkers, including sample collection, storage conditions, vesicle isolation methods, RNA extraction procedures, and data normalization strategies, can substantially affect reproducibility and comparability across studies. Further research is needed to standardize protocols, validate candidate biomarkers in large cohorts, and determine if EV-derived biomarkers add enough improved diagnostic performance over free-circulating sncRNAs to justify the added complexity and cost of their analysis. This emerging area warrants more specific investigation and could serve as the basis for future reviews focused specifically on the clinical potential of EV-associated sncRNAs for the detection of advanced colorectal neoplasia and barriers for their translation to clinical practice.

## 4. Final Considerations and Future Directions

**Figure 2:**
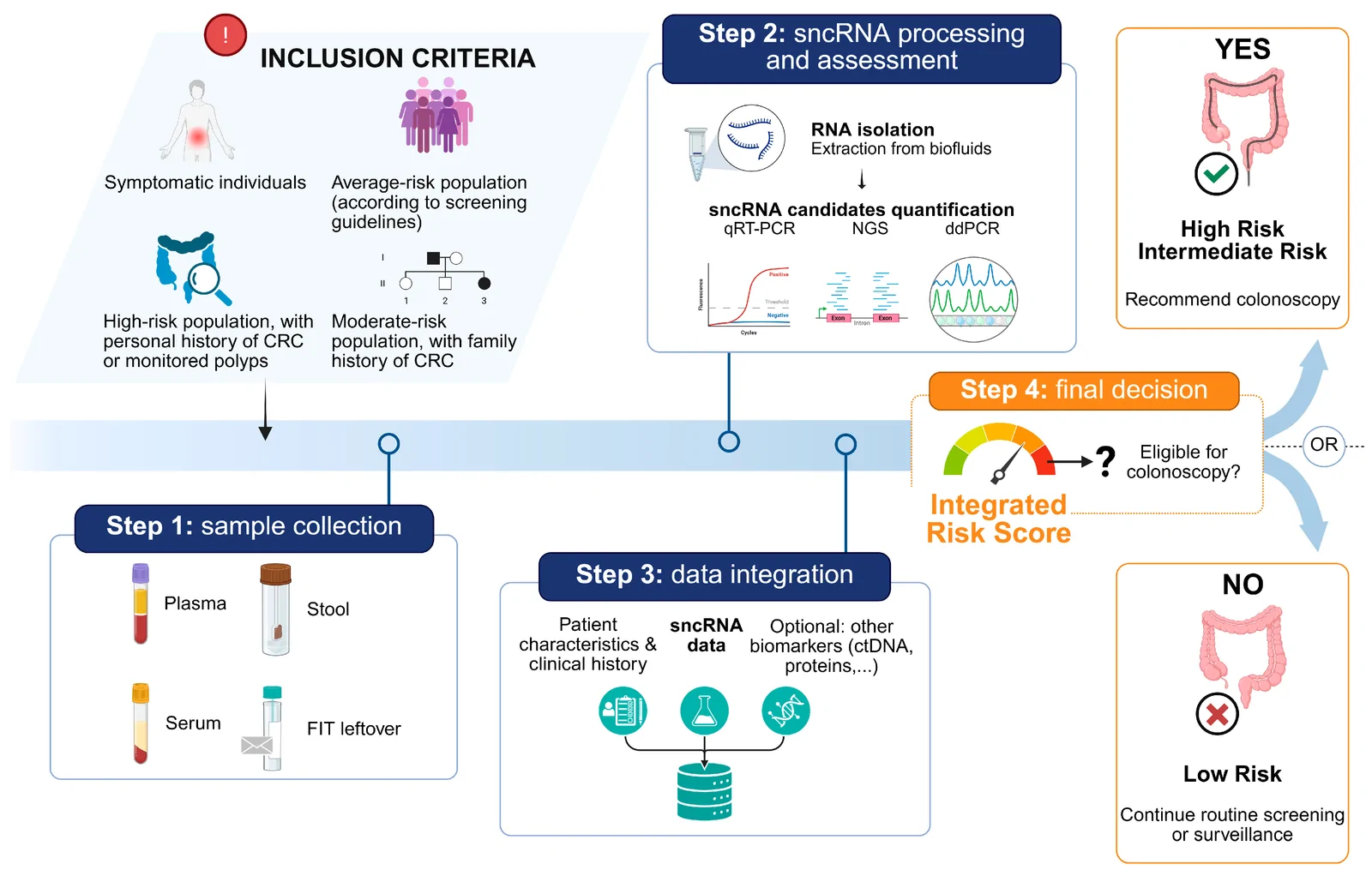
Schematic workflow that illustrates the different steps for the suggested clinical application of sncRNA-based biomarkers in the detection of advanced colorectal neoplasia. Created in BioRender. Di Battista, C. (2026) https://BioRender.com/2m965g5.

CRC is a preventable and curable disease when it is diagnosed as premalignant lesions or even at early stages. Indeed, patients who are diagnosed with premalignant lesions or well-localized tumors can undergo more effective and less aggressive treatments, making them less prone to long-term side effects and relapses. This highlights the importance of improving current screening strategies, with the objective of reducing CRC incidence and, ultimately, mortality. Different CRC screening programs are currently used in clinical practice. Colonoscopy is the gold standard technique for the detection of advanced adenomas and CRC tumors. However, it is still associated with high costs, complications, and patient discomfort. Other CRC screening techniques applied are stool-based tests, which have low sensitivity and specificity, especially regarding the detection of premalignant lesions. This eventually results in a high rate of false positive tests and unnecessary confirmative colonoscopies. Therefore, the optimization and implementation of new non-invasive tools to detect advanced colorectal lesions is a pending clinical need. The detection of sncRNAs as biomarkers for CRC stood out as a promising strategy, since studies showed that their presence in body fluids (mainly in blood and stool) can reflect the presence of CRC tumors at early stages, and even premalignant lesions (Figure 2). However, validations in larger and independent cohorts are still missing, as well as formal comparisons with gold standard techniques for CRC diagnosis. Moreover, there are technical limitations that still need to be overcome. Thus, the standardization of pre-analytical and analytical procedures, along with the consideration of sources of biological heterogeneity that may affect circulating sncRNA signatures, will be key to validating biomarkers for clinical implementation. Finally, there remains a need for more biomarkers capable of detecting premalignant lesions, since only a few studies have addressed this issue. Given the well-characterized course of events that lead to CRC, finding biomarkers with high sensitivity and specificity to detect premalignant lesions would allow the excision of advanced polyps and would prevent the onset of CRC. Although encouraging results have been reported, it is still possible to improve the characterization of sncRNAs as biomarkers for advanced colorectal lesions. Growing evidence suggests that the combination of different sncRNAs into signatures or integration with other molecular markers (e.g., ctDNA, methylation markers, and protein markers) or clinical parameters (e.g., FIT value, age, and sex), rather than the use of single molecules, will be key to fostering clinical implementation of biomarkers. Artificial intelligence and other computational tools that enable the integration of multilayer models will be essential for identifying the optimal combinations that most efficiently predict the presence of advanced colorectal neoplasia in large independent cohorts, while minimizing the risk of overfitting, particularly in the validation of biomarkers in CRC screening populations, where a relatively small number of events is expected.

**Methodology:** This review has been conducted using a structured, criteria-driven approach to identify relevant studies of the role of sncRNAs in body fluids for early detection of CRC. Eligible publications were peer-reviewed articles, indexed in the PubMed database, that analyzed human patient samples and provided clear methodological descriptions. Only studies including at least 40 CRC cases were considered. The literature search was performed using combinations of the following terms: “colorectal cancer”, “screening”, “early detection”, “miRNA”, “sncRNA”, “circulating biomarkers”, “blood”, and “feces”. Studies published between 2008 (corresponding to the first reports describing circulating miRNAs in blood_ and the date of manuscript preparation were evaluated.

## Figures and Tables

**Table 1:** Main species of small non-coding RNAs.

sncRNA	Abbreviation	Size (nt)	Main Functions
Micro RNA	miRNA	20–24	Transcriptional regulation of protein-coding genes by modulation of mRNA translation.
PIWI-interacting RNA	piRNA	24–32	Silencing of transposable elements at the transcriptional and post-transcriptional level and regulation of protein-coding genes by modulation of mRNA translation.
Transfer RNA	tRNA	70–93	Adaptors for ribosomal protein synthesis. Regulation of protein translation by multiple mechanisms.
Small nucleolar RNA	snoRNA	60–300	Processing and post-transcriptional modification of rRNA. Regulation of alternative splicing and transcriptional regulation by modulation of mRNA translation.

## Data Availability

No new data were created or analyzed in this study. Data sharing is not applicable to this article.

## Abbreviations

AA, advanced adenoma; AUC, area under the curve; CEA, carcinoembryonic antigen; CRC, colorectal cancer; CTC, circulating tumor cell; ctDNA, circulating tumor DNA; ctRNA, circulating tumor RNA; ddPCR, droplet digital PCR; DNA, deoxyribonucleic acid; FIT, fecal immunochemical test; FOBT, fecal occult blood test; IBD, inflammatory bowel disease; miRNA, micro RNA; mRNA, messenger RNA; NAA, non-advanced adenoma; NGS, next-generation sequencing; PCR, polymerase chain reaction; piRNA, PIWI-interacting RNA; RNA, ribonucleic acid; ROC, receiver operating characteristic; RT-qPCR, real-time quantitative PCR; sncRNA, small non-coding RNA; snoRNA, small nucleolar RNA; tRF, tRNA-derived fragment; tRNA, transfer RNA.
